# Emerging strategy towards mucosal healing in inflammatory bowel disease: what the future holds?

**DOI:** 10.3389/fimmu.2023.1298186

**Published:** 2023-12-14

**Authors:** Min Wang, Jingyan Shi, Chao Yu, Xinyi Zhang, Gaoxin Xu, Ziyan Xu, Yong Ma

**Affiliations:** ^1^ Department of General Surgery, Nanjing First Hospital, Nanjing Medical University, Nanjing, China; ^2^ Medical School, Nanjing University, Nanjing, China

**Keywords:** inflammatory bowel disease, mucosal healing, intestinal mucosal barrier, emerging strategy, organoid

## Abstract

For decades, the therapeutic goal of conventional treatment among inflammatory bowel disease (IBD) patients is alleviating exacerbations in acute phase, maintaining remission, reducing recurrence, preventing complications, and increasing quality of life. However, the persistent mucosal/submucosal inflammation tends to cause irreversible changes in the intestinal structure, which can barely be redressed by conventional treatment. In the late 1990s, monoclonal biologics, mainly anti-TNF (tumor necrosis factor) drugs, were proven significantly helpful in inhibiting mucosal inflammation and improving prognosis in clinical trials. Meanwhile, mucosal healing (MH), as a key endoscopic and histological measurement closely associated with the severity of symptoms, has been proposed as primary outcome measures. With deeper comprehension of the mucosal microenvironment, stem cell niche, and underlying mucosal repair mechanisms, diverse potential strategies apart from monoclonal antibodies have been arising or undergoing clinical trials. Herein, we elucidate key steps or targets during the course of MH and review some promising treatment strategies capable of promoting MH in IBD.

## 1 Introduction

Inflammatory bowel disease, a chronic and recurrent gastrointestinal disorder encompassing Crohn’s disease (CD) and ulcerative colitis (UC), is characterized by non-specific inflammation of the intestinal mucosa (1). The vast majority of IBD patients experience cycles of recurrence and remission marked by abdominal pain, diarrhoea, fever, and tenesmus (2, 3). The natural history of IBD is highly individualized, which varies across disease stages, ranging from asymptomatic or mild disease to severe manifestations necessitating hospitalization, surgery, disability, or even mortality (4). Most patients with IBD can achieve long-term symptom control through pharmacological therapy alone (5). However, in most cases where drug therapy fails to adequately suppress intestinal inflammation or when complications such as obstruction, perforation, and bleeding arise, surgical interventions are often indispensable to remove the affected intestine (6, 7). In turn, intestinal resection significantly impacts patients’ postoperative quality of life and may result in complications such as anastomotic leakage, bleeding, and short bowel syndrome, which could cause severe gastrointestinal damage or even systemic dysfunction (8). Though several treatment methods are available, unfortunately, IBD cannot be cured.

**Figure 1:**
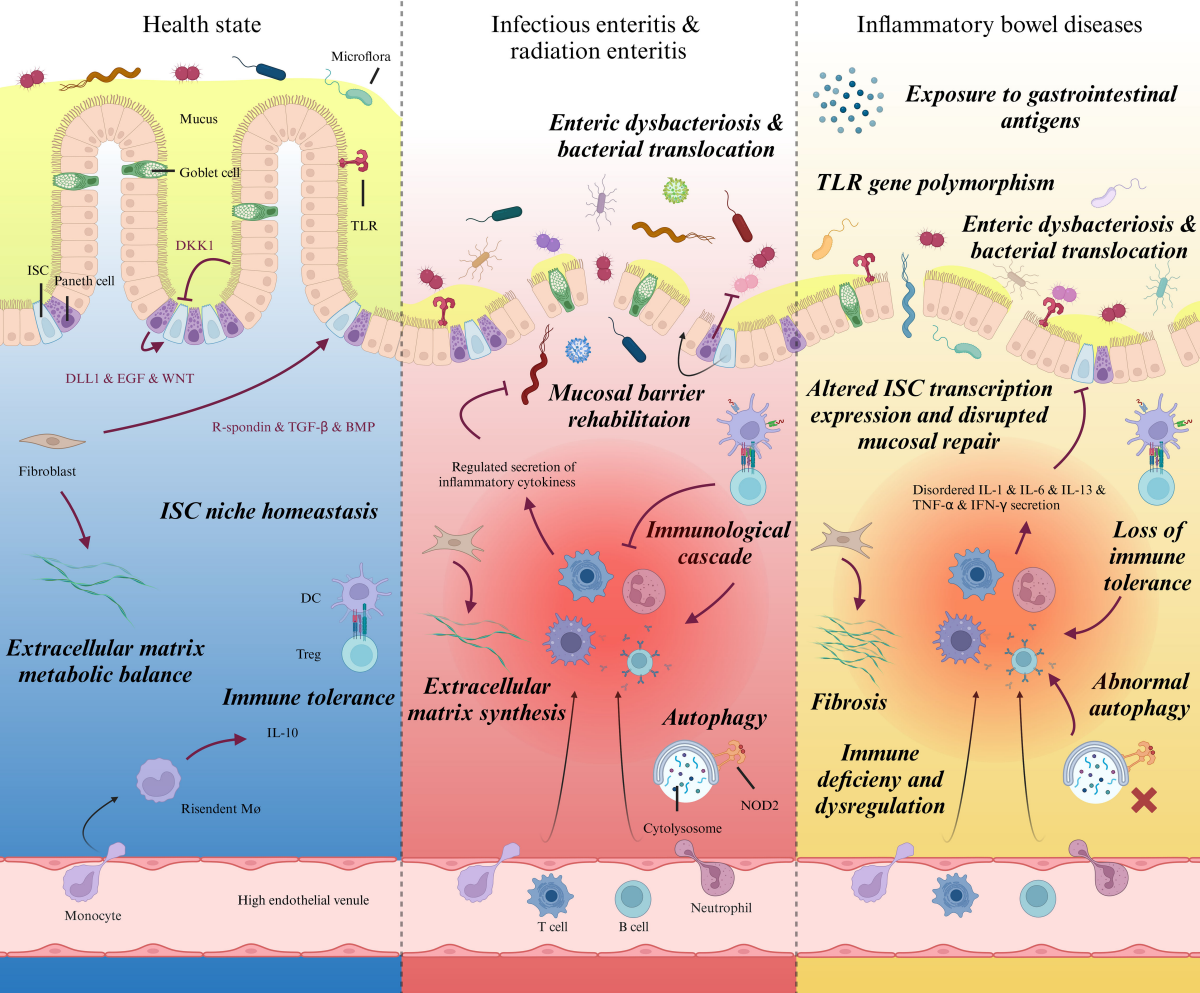
Intestinal pathophysiological characteristics during homeostasis, specific injury, and IBD. Schematic diagram showing prominent pathophysiological variations of intestinal mucosa and lamina propria in different states. Left: Gradients of biochemical signals secreted by neighboring Paneth cells, fibroblasts and enterocytes regulate the self-renewal and differentiation of ISCs synergistically. By programmed proliferation, differentiation, and migration towards top of the villi, ISC plays a significant role in intestinal barrier integrity and homeostasis. Middle: Epithelial cells are vulnerable to microorganism invasion or radiation damage, which usually bring about acute or subacute inflammation in lamina propria. The exposure to intestinal pathogens launches regulated immunoreaction. Upon receiving the activation from immune signal and physicochemical changes within the stem cell niche, ISCs exert stemness and produce terminally differentiated epithelial cells to replace injured ones and rehabilitate mucosal barrier. Right, Despite the vague etiology and different pathogenic site; IBD patients share common pathophysiological features, including immune dysregulation within lamina propria, enteric dysbacteriosis, fibrosis. Recently, much investigations have verified protracted post-transcriptional modification and damaged viability of ISCs as well as distinct differentiation pattern alongside the intestinal epithelium, which could be the potential immediate cause to delayed mucosal healing in IBD patients. ISC, Intestinal stem cell; DKK1, Dickkkopf-1; TLR, Toll-like receptor; EGF: Epidermal growth factor; TGF, Transforming growth factor; BMP, Bone morphogenetic protein; DC, Dendritic cell; Treg, Regulatory T cell; NOD2, Nucleotide-binding oligomerization domain 2.

Various elements contribute to the pathophysiology of IBD (**Figure 1**). In the interplay of various intricate factors, a cascade of events occurs, resulting in modified microbial communities, aberrant expression of tight junction (TJ) proteins, and impairment to the mucus layer that facilitates the infiltration of luminal bacteria into the submucosa (9, 10), leading to mucosal inflammation and destruction. Subsequently, neutrophils are recruited to the site of infection, where phagocytose microorganisms and generate neutrophil extracellular traps (NETs) to immobilize pathogens (11). The demand for neutrophils during acute inflammation is met by rapid granulopoiesis in the bone marrow driven by the IL17-IL23A-CSF3 axis (12). Monocytes and macrophages eliminate cellular debris generated by neutrophil activity, producing TNF, IL-6, IL-12, and IL-23 (2, 13). Subsequently, monocyte-macrophages act as antigen-presenting cells (APC) and participate in T helper cell differentiation into Th1, Th2, Th17, and other subtypes (14). B cell activation occurs in IBD, and the expansion of B cells has been shown to impede epithelial-stromal cell interactions (15). Further comprehension of the pathogenesis of IBD is pivotal in propelling research and development of targeted pharmaceuticals and novel therapeutic approaches, thereby facilitating the treatment and management of IBD while enhancing the quality of life for patients afflicted with this condition.

As the etiology of IBD remains elusive, the primary objective of treatment is to alleviate inflammation through various therapies including 5-aminosalicylic acid (5-ASA), antibiotics, corticosteroids, immunosuppressive agents and biological interventions (16). In the past, symptom assessment remains the cornerstone of clinical practice in the diagnosis and management of IBD, playing a pivotal role in evaluating disease severity, surgical candidacy, and treatment response (17). This underscores the importance of adopting a symptom-driven approach to managing symptoms such as abdominal pain, diarrhea, gastrointestinal bleeding, among others (17, 18). Ultimately, achieving clinical remission represents a key therapeutic goal for most IBD patients, which is accepted by patients and doctors. The utilization of standardized clinical scoring systems, such as the Truelove and Witts criteria, facilitated a more objective evaluation of the disease (19). The resolution of intestinal inflammation, repair of intestinal damage, and restoration of intestinal ecological balance should not be solely inferred from the relief of clinical symptoms (20). Currently, mucosal healing has emerged as a new long-term goal in IBD treatment and has garnered significant attention in recent years (21, 22).

## 2 Definition of Mucosal healing

A crucial characteristic of IBD is the impairment of the intestinal mucosal barrier (IMB) (23), which leads to the translocation of microorganisms and other antigens into the internal environment, resulting in uncontrolled immune activation (24). Current studies believe that the impairment of IMB may be the initial link in the development of IBD, whose severity is closely related to the symptoms (23). The foundation of mucosal healing is based on an undamaged barrier that limits the movement of bacteria and subsequent immune response activation (25). Definitions specific to mucosal healing often rely on endoscopic criteria such as the absence of ulcers with no fragility, blood, erosion or ulceration in CD and UC; or complete resolution of inflammatory and ulcerative lesions in both forms of IBD (25, 26). The evaluation criteria of mucosal healing are constantly changing, but Simplified Endoscopic Activity Score for Crohn’s Disease (SES-CD) and Mayo score based on endoscopy is still one of the mainstream methods of mucosal healing diagnosis (27) (28). In fact, microscopic inflammation has been reported in up to 25% of patients with endoscopic mucosal healing (29). Additionally, the histological evaluation of mucosal healing is constantly evolving, and the histological definition and criteria of mucosal healing have evolved from “elimination of mucosal ulcer/erosion” to “absence of neutrophilic infiltration” (30). Therefore, the presence or absence of active inflammation has become a consensus in histological evaluation of mucosal healing. Currently, more than 30 different histological scoring systems have been created (31, 32), despite the fact that their utilization in clinical settings is still restricted. The Simplified Histologic Mucosal Healing Scheme (SHMHS) can identify active inflammation that may not be visible during endoscopy, and facilitate clinical application (33). Some scholars integrated endoscopy, histology, and other factors to avoid the potential inaccuracies of single index diagnosis. They focused on assessing neutrophil infiltration as a key determinant of disease progression and histological remission (34). In fact, the diagnosis of mucosal healing based on endoscopic techniques faces significant challenges in clinical practice (35, 36). For certain IBD patients, endoscopy is neither tolerable or necessary (37). Therefore, it is worth exploringto balance the advantages and disadvantages of endoscopy or expand the definition of mucosal healing further while avoiding invasive interventions. Some scholars have suggested that fecal calprotectin and other markers can serve as substitutes for serum or fecal markers (38–40). This approach would alleviate the discomfort experienced by IBD patients and reduce treatment costs (41). In the past few years, the application of artificial intelligence (AI) has mitigated operator errors in endoscopy or pathological interpretation and optimized diagnostic criteria for mucosal healing (42, 43). It is foreseeable that mucosal healing evaluation will need to evolve towards a multifactorial approach.

The process of mucosal healing involves a complex interplay between ISCs and signaling molecules throughout the course of regeneration. The regulation of ISC-driven differentiation into epithelial cells is mediated by BMP, Wnt, and Notch signalling pathways (44, 45). It is widely accepted that intestinal epithelial cells (IECs) located at the border of damaged mucosa, regulated by transforming growth factor-alpha/beta (TGF-α/β), trefoil factor and other signaling molecules (46–48), undergo redifferentiation, lose their columnar phenotype and acquire a migratory phenotype to migrate towards the defect site in order to form a primary barrier (49). Subsequently, regulated by nuclear factor-κb (NF-κB) and other factors (50), epithelial cells facilitates the stabilization and maturation of the nascent mucosal barrier. Ultimately, these newly formed undifferentiated epithelial cells undergo a process of differentiation, giving rise to a diverse array of mature intestinal epithelial cell types, encompassing Paneth cells, goblet cells, enteroendocrine cells, enterocytes and M cells, which give rise to the intestinal epithelial barriers (IEB).

## 3 The progress and dilemma of traditional therapies

When considering the possibility of incomplete healing of a patient’s intestinal mucosa, how to repair the intestinal ecosystem becomes a crucial question. Currently available treatments, such as sucralfate, 5-ASA, antibiotics, corticosteroids, and immunosuppressive agents (51, 52), primary focus symptom alleviation and mitigating of chronic inflammation rather than mucosal healing and intestinal ecological restoration as the end point of treatment. Although corticosteroids, methotrexate, and some immunosuppressive drugs promote healing of the intestinal mucosa to some extent in some studies (53, 54), they also lead to an increased risk of infection and cancer due to immunosuppression (55), so clinicians must balance the benefits with the risks.

Through continuous exploration of the complex mechanisms underlying IBD, treatment based on precise molecular targeting of inflammatory cascades has greatly advanced IBD therapy from clinical symptom relief to mucosal healing. Since the development and application of anti-TNF-α, a variety of monoclonal antibodies have been continuously developed and applied to enrich the precision targeted therapy for IBD.As first-line agents for the treatment of IBD, anti-TNF-α monoclonal antibodies such as infliximab and adalimumab have long been utilized to mitigate inflammation and promote mucosal healing (56). Anti-TNF-α therapy targets proinflammatory cytokines by obstructing soluble and membrane-bound TNF-α, inducing direct and indirect apoptosis of TNF-α-producing T cells and macrophages (57). Ultimately regulating intestinal inflammation, restoring the integrity of the IEB, and promoting mucosal healing. Several clinical studies have established a correlation between serum anti-TNF drug levels and mucosal healing in patients. For instance, Bella Ungar et al. (58) reported that maintaining a serum infliximab level of 6-10μg/mL can result in mucosal healing in 80% to 90% of adult IBD patients.

In certain patients, anti-TNF-α therapy may exhibit either non-response or withdrawal response, which could be attributed to specific network connections among IgG plasma cells, inflammatory mononuclear phagocytes, activated T cells and stromal cells (59). For such individuals, treatment alternatives have shifted towards monoclonal antibodies that target other inflammatory factors. For example, the monoclonal antibody ustekinumab, which specifically targets IL-12/23p40, demonstrated a significant reduction in endoscopic range of motion among patients with IBD, thereby exhibiting its capacity to facilitate the restoration of mucosal integrity (60). Risankizumab, guselkumab, and mirikizumab are inhibitors of IL-23p19 that have demonstrated efficacy in clinical trials for symptom improvement, endoscopic findings enhancement, and histological remission (61–63). Additionally, the specific target of vedolizumab is the α4β7 integrin, which interacts with cell adhesion molecule 1 (MAdCAM-1). In a long-term study conducted on 374 individuals diagnosed with UC, vedolizumab treatment resulted in clinical remission and mucosal healing for more than half of the participants (64). In recent years, a plethora of novel oral small-molecule drugs have emerged, including the JAK inhibitors tofacitinib, filgotinib, and upadacitinib as well as the sphingosine-1-phosphate (S1P) receptor modulator ozanimod, which have been granted regulatory approval for treating IBD (65, 66). Limited clinical trials have unequivocally confirmed their potential to foster mucosal healing (66, 67). Currently, an array of monoclonal drugs are being actively developed with promising available clinical data (65–67).

Anti-tumor necrosis factor-alpha (TNF-α) drugs have ushered in a new era of IBD treatment (68). At present, the use of monoclonal antibodies as a treatment for IBD presents a novel therapeutic approach that yields favorable outcomes for the majority of patients (69, 70). Nevertheless, some individuals remain unable to achieve optimal results despite this intervention, and clinicians continue to grapple with issues related to antidrug antibody-mediated ineffectiveness and withdrawal reactions (55, 71, (72). Additionally, physiological inflammation serves as a self-protective mechanism of the body; however, excessive immune response inhibition may result in other risks such as infection. The intricate involvement of proteins and signaling pathways in IBD highlights the significance of individual differences and remains a significant field for further investigation (73, 74).

## 4 Novel and potential emerging therapies

**Figure 2:**
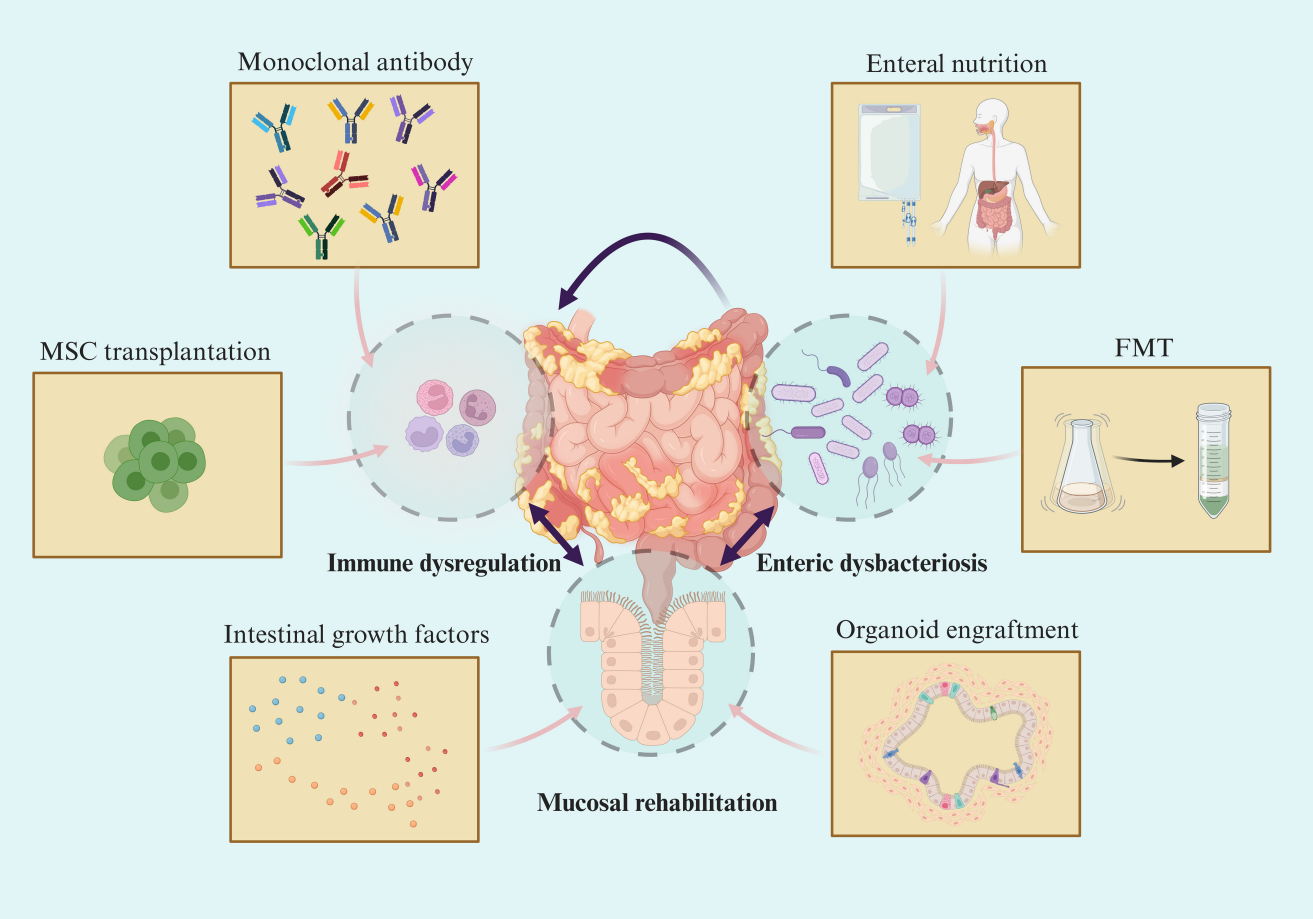
Novel therapeutic strategy towards mucosal healing in IBD. Symptoms in IBD patients are highly correlated with the degree of mucosal damage. Although emerging therapies target different intestinal components, they are beneficial to mucosal barrier rehabilitation and mucosal healing via intertwined pathophysiological changes in IBD.

In recent years, there has been continuous development of drugs for the treatment of IBD and significant progress in therapeutic strategies. However, current treatment methods such as 5-ASA, monoclonal antibodies, and other drugs have not yet achieved optimal results (51). For some patients, these treatments may even lead to serious complications. Most of the current treatment methods are based on the “immunity” strategy, and exploring approaches to mitigate immune suppression may present a novel concept, as immunity is indispensable for maintaining human health. While some of these methods may seem impractical for clinical implementation, others such as enteral nutrition (EN), faecal microbiota transplantation (FMT), and the utilization of certain growth factors have been partially integrated into clinical practice (75–77). These approaches demonstrate their potential in promoting mucosal healing without immunosuppression. These novel treatment methods have significantly advanced the development of intestinal mucosal healing and even hold the potential for curing IBD (**Figure 2**).

### 4.1 Dietary management and enteral nutrition

Dietary factors influence the occurrence and progression of IBD. Several epidemiological studies have confirmed the association between dietary macronutrient and micronutrient intake and the pathogenesis of IBD. For instance, adhering to a dietary pattern rich in fruits, vegetables, and fish has been shown to reduce the risk of developing UC by 50% among high school students (78). The role of diet in IBD is further supported by advancements in understanding genetic architecture and the gut microbiome’s influence on immune dysregulation leading to gut inflammation. However, parenteral nutrition (PN) adversely affects intestinal growth, reducing mucosal mass, cell proliferation, and mucosal immune function, which undoubtedly worsens the condition of IBD. Diet can impact gut inflammation through various mechanisms including modulation of the microbiome, tight junctions, and mucosal barriers. Dietary interventions that alter a patient’s microbiome composition during remission have demonstrated the potential for reversing many features associated with active diarrhea by affecting its metabolic function alongside its composition (79). A range of dietary approaches have been developed for patients with IBD, including Crohn’s Disease Exclusion Diet, IBD Anti-Inflammatory Diet, Specific Carbohydrate Diet, and others (80–82). These diets commonly emphasize a high intake of fruits and vegetables, and lean meats, while excluding most dairy products, canned foods, tea, coffee, and alcohol. However, they exhibit variations in recommendations regarding certain foods such as yogurt, beans, and nuts. The aforementioned also implies the necessity for further research into the impact of alterations in individual dietary components on the human physique and the development of more suitable dietary patterns. Moreover, the intricacy involved in food handling, processing, and packaging exacerbates the heterogeneity observed in dietary intervention studies (83). Consequently, finding a solution to effectively achieve homogeneity comparison becomes an issue that demands resolution.

The efficacy of enteral nutrition treatment modalities has been partially demonstrated, with exclusive enteral nutrition (EEN) serving as a primary therapeutic approach for pediatric patients with CD, effectively alleviating clinical symptoms and promoting mucosal healing (84). Clinical studies have demonstrated that over 70% of children with CD treated with EEN achieved mucosal healing (85, 86). However, in adult patients, poor palatability often leads to low compliance with EEN, thereby diminishing its therapeutic efficacy (87). To assess the feasibility of using EEN in adult patients, Wall et al. (88) employed EEN alone or in combination with partial enteral nutrition for individuals suffering from active CD. The results showed clinical remission, reduced levels of serum CRP and FC, as well as increased serum insulin-like growth factor 1 (IGF-1) levels, suggesting a potential treatment for achieving mucosal healing. In another study conducted by Yang et al. (89), 47% of patients with complex active CD attained endoscopic mucosal healing following EEN treatment. Although not commonly used in UC patients, available evidence confirms the viability of synergistic corticosteroid therapy in cases of acute severe UC, significantly reducing CRP and FC levels (90). When attempting to elucidate the mechanism underlying mucosal healing induced by exclusive EEN, studies have observed a reduction in intestinal flora diversity, which appears contradictory to treatment expectations (91, 92). Alternative explanations encompass the modulation of inflammatory factors and hormones by EEN, including the secretion of serum IGF-1 and TGF-β1, as well as the promotion of mucosal healing (93). The future research directions should primarily focus on elucidating the underlying mechanisms by which EN enhances mucosal healing and its differential effects in patients with UC and CD. The forthcoming endeavors hold great promise in unraveling the intricate principles of enteral nutrition through extensive multicenter randomized trials, a pursuit that carries immense significance. Furthermore, enhancing palatability of EEN may facilitate wider adoption among adult patients.

### 4.2 Organoid culture engraftment

Organoids, derived from stem cells through *in vitro* culture and self-assembly, represent three-dimensional (3D) cell cultures that possess both structural and functional specificity akin to the corresponding tissues (94). The organoids formed by ISCs *in vitro* usually form Bud-like organs composed of hyperplastic protruding cryptlike domains, closely resembling the structure of intestinal epithelium *in vivo* (95). In 2009, Clevers and Sato first reported a method for long-term culture of intestinal epithelium from purified stem cells (96). Intestinal organoids have made many breakthroughs in basic research and personalized treatment development for IBD with the mature application of stem cell *in vitro* 3D culture technology. Intestinal organoids are used for disease modelling and as preclinical tools in regenerative medicine through *in vivo* transplantation (97). Multiple studies have demonstrated the possibility of transplanting organoids of intestinal stem cells into damaged colons to lead to tissue regeneration and mucosal healing (98, 99). Back in 2012, a study presented a comprehensive account of epithelial organoid transplantation into the colon of a mouse model with inflammatory enteritis. In this experiment, organoids were administered via enema and exhibited effective adherence to the injured site, thereby facilitating epithelial restoration (100). Previous studies also demonstrated that intestinal organoids implanted into dextran sulfate sodium (DSS) -induced colitis models can accurately target donor cells to locate on the surface of colitis-induced ulcers and begin to rebuild and repair crypt structures (101). Consistent with these findings, the same mouse-derived intestinal organoids were injected anally by Watanabe and colleagues to facilitate colon repair in UC mice. These cultured organoids exhibited precise targeting within the damaged intestinal epithelium, as anticipated based on prior developmental investigations, these organoids retained their regional characteristics and exhibited functional enteric properties (102). The rapid development of endoscopic techniques for organoid infusion has now been shortened to 10 minutes (102). However, there remain numerous challenges to be addressed in organoid transplantation. It is yet to be determined whether the success of organoid cultures in non-IBD tissues can be replicated in IBD tissues, and whether autologous transplantation offers advantages over allogeneic transplantation. Additionally, evaluating the stability of cultured karyotypes is essential to understand the risk of tumorigenesis associated with these techniques (98). Furthermore, there is a need for improvement in the current protocols utilized for the isolation and cultivation of intestinal organoids. Studies have shown that intestinal organoids may not develop appropriately when derived from inflamed segments with damaged or lost epithelial layers (103). Nonetheless, the concept of cultivating new tissue *in vitro* and subsequently transplanting it into areas devoid of mucosa holds great promise for the future.

### 4.3 Mesenchymal stem cells transplantation

Mesenchymal stem cells (MSCs) are thought to be a versatile type of stem cells capable of differentiation, which can be derived from various sources, such as bone marrow, adipose tissue, umbilical cord and so on (104). MSCs have demonstrated the capacity to relocate towards areas affected by colitis-induced damage, where they possess the ability to differentiate into constituent cells of the intestinal epithelium or vascular endothelium. Alternatively, they can work together with intestinal epithelial stem cells in producing cytokines and promoting the healing process of the mucosal layer (105). MSCs mediate intestinal immune regulation through the secretion of prostaglandin E2 (PGE2), hepatocyte growth factor (HGF) and nitric oxide (NO) (106). Due to the presence of these soluble factors, MSCs exhibit immunomodulatory effects on various immune cell subsets. It has been documented that MSCs suppress Th1 and Th17 responses while promoting Th2 and Treg-mediated responses, thereby ameliorating colonic inflammation (107). Furthermore, MSCs supported intestinal barrier function by inducing the proliferation of IECs and up-regulating expression of TJ proteins, thereby ensuring intestinal barrier integrity (108). Hence, the transplantation of MSCs emerges as a promising therapeutic strategy for IBD due to their ability to secrete diverse bioactive molecules. Various clinical trials are currently underway to assess the safety and efficacy of MSCs in patients with IBD, and these findings have yielded positive outcomes in animal studies. Although the safety and short-term effectiveness of MSCs administration has been demonstrated, further validation is required to ascertain their long-term efficacy in transplantation. Prior research has shown that local injection of adipose-derived MSCs can effectively treat fistulas in CD (109, 110), and the recent meta-analysis encompassing seven trials investigating the efficacy of MSCs derived from bone marrow and umbilical cord in patients with UC demonstrated a certain level of efficacy (111). Despite some positive findings, the outcomes of systemic MSCs therapy in luminal CD patients thus far have been underwhelming. The results of various clinical trials have exhibited significant heterogeneity to date (112, 113), emphasizing the necessity for high-quality randomized controlled clinical trials and fundamental research.

In fact, less than 1% of intravenous MSCs can reach the damaged colon (114), and the method of MSCs transplantation is controversial. Despite the non-immunogenicity of allogeneic and autologous MSCs (115), stem cell transplantation may present certain drawbacks, including significant financial costs and the possibility of malignant transformation (112). Furthermore, the safety profile of systemic MSCs remains to be thoroughly investigated due to reports of exacerbated outcomes in patients with UC or CD, necessitating further exploration. Additionally, it is imperative to address the optimal source, route of administration, and dosage of MSCs.

Some studies have turned their attention to exosomes secreted by mesenchymal stem cells (MSC-Exos) and have been used in various IBD models with promising results (116). Yang et al. (117) have significantly improved IBD symptoms by intraperitoneal injection of MSC-Exos. MSC-Exos can alleviate intestinal inflammation by increasing the expression of tumor necrosis factor-stimulating gene 6 (TSG-6) expression to repair the IMB and maintain immune balance (117). The utilization of MSCs for regenerating damaged mucosa holds great promise in expanding both the scope and efficacy of this therapeutic approach. A primary limitation of MSC-Exos therapy lies in its low yield, which presents a significant impediment to its clinical application. However, this challenge can be effectively overcome by embracing a 3D culture system as an alternative to the conventional 2D culture system (118).

The therapeutic approaches for MSCs can be complex and varied, incorporating autologous application, allogeneic MSCs, and cellular derivatives. These advancements hold immense potential in paving the path towards groundbreaking therapeutic strategies in IBD. Nevertheless, attaining this goal requires tackling fresh challenges by skillfully integrating cutting-edge methodologies and technologies while judiciously selecting ideal sources of MSCs to specifically target the multifaceted pathophysiological mechanisms involved in IBD.

### 4.4 Fecal microbiota transplantation (FMT)

Intestinal dysbiosis plays a pivotal role in the pathogenesis and progression of IBD, as well as in the persistence of complications that significantly impact patients’ prognosis and overall quality of life (119). Research findings have indicated a notable decrease in the prevalence of advantageous microorganisms, including *Bifidobacterium*, *Lactobacillus* (Enterobacter), and *Firmicutes*, among IBD patients (120, 121). Conversely, the abundance of pathogenic intestinal bacteria, such as *Escherichia coli* or *Salmonella typhimurium*, is significantly increased, which facilitates the progression of chronic mucosal inflammation by disrupting the integrity of the IMB (122–124). FMT, also known as the transfer of healthy individuals’ fecal microbiota to the intestinal tract of patients with IBD, presents a novel approach for treating IBD (76). The administration of FMT is commonly performed through nasogastric or nasojejunal tube, colonoscopy, enema, or oral capsule delivery routes (125). FMT can increase the diversity or abundance of the intestinal flora of the recipient. Specific microorganisms and microbial metabolites can regulate the wound repair of colonic epithelium after mucosal injury (126–128), thereby relieving clinical symptoms and promoting mucosal healing. Biao et al. (129), through repeated FMT combined with partial enteral nutrition, demonstrated improved clinical symptoms and enhanced mucosal healing in pediatric patients with active CD. However, severe IBD patients often experience adverse consequences due to compromised intestinal mucosal barrier function when undergoing FMT (130, 131). A systematic review of 129 studies on FMT across various medical conditions revealed an overall incidence rate of adverse events (ADE) at 19%, encompassing symptoms such as abdominal pain, diarrhea, fever, and other gastrointestinal disorders (132). In future research, greater attention should be devoted to aspects such as fecal donor selection, delivery system optimization, treatment duration determination, and standardization of emphasis in the field of fecal transplantation. The infection caused by Clostridium difficile (CDI) is a prevalent complication of IBD and is closely linked to the unfavorable prognosis of IBD. Recently, the Food and Drug Administration (FDA) has granted approval for rectal administration of Live-JSLM (REBYOTA) and oral delivery of Vowst, both microbiota-based products, for the treatment of CDI (133, 134). However, clinical trial evidence that excludes patients with IBD and uses clinical symptom relief as an effective indicator for treating CDI is still insufficient to apply these drugs to patients with IBD (135). Furthermore, considering the presence of potential adverse reactions associated with these drugs, it is imperative to meticulously evaluate both efficacy and safety aspects in forthcoming clinical trials.

Short-chain fatty acids (SCFAs), as crucial metabolites of the intestinal microbiota, primarily consist of acetate, propionate, and butyrate (122). They not only facilitate the proliferation and differentiation of colonic epithelial cells, maintaining intestinal mucosal epithelial barrier stability, but also regulate gut inflammatory response (135, 136), thereby potentially serving as treatments for FMT. *In vitro* and animal studies have demonstrated that butyrate possesses anti-inflammatory properties by inhibiting the production of pro-inflammatory cytokines and chemokines, thereby alleviating inflammation during IBD progression (122, 137). This discovery holds significant implications for IBD treatment, suggesting that supplementation of short-chain fatty acids could be a promising approach to promote intestinal mucosal healing. Both oral administration and enema delivery can be employed to administer short-chain fatty acids; however, enema administration circumvents challenges associated with intestinal absorption while ensuring direct drug delivery to the colon (138). Consequently, this may lead to divergent outcomes in patients with UC and CD. In colitis-induced mouse models, acetate supplementation plays a crucial role in the gut’s response to injury and tissue repair (139). A preliminary study by Facchin et al. confirmed that sodium butyrate supplementation reduces inflammation in patients with IBD (140). Although there exists some preclinical evidence along with limited clinical data supporting potential therapeutic applications based on SCFAs therapies, further research is warranted to comprehensively elucidate their mechanisms of action, safety profiles, optimal dosage regimens, as well as long-term effects for treating IBD.

### 4.5 Extracellular matrix

The intestinal extracellular matrix (ECM) is a functional protein complex assembled in a specific grid structure composed of glycosaminoglycans (GAGs) (141, 142). Its major components include collagens, elastin, laminins and proteoglycan (143). In addition to providing structural support for cells in tissues, the ECM also plays an active role in various cellular processes such as proliferation and migration (144). An increasing body of evidence demonstrates that in the process of mucosal healing, patients with IBD experience an augmented damage and repair mechanism of extracellular matrix due to enhanced protease activity and deposition of ECM degradation products, thereby disrupting the delicate equilibrium between ECM damage and repair (145, 146). Lindholm et al. (147) found that DSS caused direct damage to the intestinal basement membrane in an acute colitis rat model, and established a robust association between Collagen III remodeling and matrix regeneration during resolution of the injury. Meanwhile, the reconstruction of the mucosal layer *in vitro* was observed to be facilitated by fibronectin and Collagen IV, thereby enhancing the migration of intestinal epithelial crypt cells (148). The study conducted by Stronati et al. (149) revealed that dipotassium glycyrrhizate (DPG) effectively facilitated the process of mucosal healing through upregulation of the expression levels of ECM remodeling enzyme PLAUR and its ligand VTN. These collectively indicate that ECM remodelling is associated with the degree of intestinal mucosal healing. Therefore, the modulation of medical pathways that impact the ECM and its regenerative capacity following injury may hold promise as potential therapeutic interventions for IBD (150, 151). In the field of tissue engineering, hydrogel formulations based on ECM have demonstrated their reparative potential in specific tissues (152). Therefore, considering the use of ECM-derived hydrogels from the gut to promote healing of intestinal mucosal injuries alone appears to be a worthwhile consideration. However, due to the intricate composition of ECM and its associated pathophysiological processes that remain incompletely elucidated, in addition to the increasing integration of ECM as a carrier and other technologies in current studies (153), challenges arise when confirming the independent role of ECM in intestinal repair, necessitating further discussion.

### 4.6 Intestinal growth factors

TGF-β is a multifunctional cytokine synthesized by various cell types, which exerts pivotal regulatory effects on diverse cellular processes, immune responses, intestinal epithelial cell proliferation inhibition, and differentiation induction (154). In the context of intestinal immunity, TGF-β serves to suppress inflammatory responses elicited by luminal bacterial antigens and attenuate the production of pro-inflammatory cytokines (155, 156). The efficacy of TGF-β treatment in ameliorating methotrexate-induced intestinal mucositis in rats has been substantiated by studies, which revealed its ability to augment p-ERK and β-catenin-mediated intestinal cell proliferation while concurrently inhibiting apoptosis and preventing mucosal injury (157). Activation of the Smad pathway mediated by TGF-β can promote mucosal injury repair through enhanced epithelial revascularization while driving processes such as intestinal fibrosis, angiogenesis, and obstruction (158). High levels of Smad7 are intracellular inhibitors of TGF-β/Smad signaling (156). It has been reported that oral administration of Smad7 antisense oligonucleotide restores TGF-β signal transduction in the intestinal tract, thereby promoting clinical symptom resolution and endoscopic healing in patients with CD (159, 160). Despite these promising findings, a Phase 3 clinical trial yielded no clinical or endoscopic efficacy for Mongersen. However, the underlying reasons for this observed ineffectiveness remain uncertain, encompassing potential factors such as inadequate concentrations of the drug in colonic or ileal tissues, intended pharmacological mechanisms, or characteristics of the patient population (161). Although no definitive evidence exists, recent research suggests that variations in diastereoisomer content across different batches of mongersen used during the development program may contribute to disparate outcomes observed in clinical trials, thus explaining the failure of the Phase 3 trial (162). Further research and exploration are required to determine whether specific modifications in manufacturing schemes can effectively reduce diastereoisomer complexity. Moreover, the study revealed a significant association between long-term Smad7 deficiency and heightened progression of intestinal fibrosis (163). It is evident that additional experiments are warranted to further investigate the role of Smad7 in inflammatory responses related to IBD and reassess the efficacy and potential risks of mongersen as a therapeutic approach for IBD.

The Trefoil factor (TFF) family comprises three peptides, known as TFF1, TFF2, and TFF3, which are released by goblet cells located in the mucosa of the intestines (164). Among these peptides, TFF3 is predominantly synthesized by goblet cells in both the small and large intestine (165). Notably, TFF3 plays a crucial role in upholding the integrity of the IMB through its regulation of cytokine expression and immune cell migration (164), and PI3K/Akt signaling pathways are activated to enhance wound healing *in vitro* (166). An evident upregulation of serum TFF3 is observed in IBD patients and confirmed to be associated with disease activity, indicating its potential as a non-invasive marker (167). Studies have demonstrated that the utilization of TFFs facilitates the restoration of gastrointestinal mucosa, thereby presenting an innovative method for addressing the management of IBD. However, conflicting outcomes are frequently observed in DSS-induced colitis animal models due to variations in TFF forms, dosages, and routes of administration (168–170). In order to optimize the application of TFFs, a recombinant adenovirus vector was constructed to deliver the human intestinal trefoil factor (hITF) gene for improved healing of intestinal mucosal injury (171). The optimal dosing strategy and underlying signaling pathways of TFF3 remain unclear; however, TFF3 has demonstrated significant potential in both the diagnosis and management of IBD.

## 5 Conclusion

With the further development of the definition of mucosal healing, the requirements of mucosal healing are no longer limited to the repair of the intestinal barrier under endoscopy and the restoration of intestinal function but require the balance and homeostasis of the intestinal ecosystem in terms of omics and biology, that is the dynamic balance between the intestinal barrier and intestinal flora. Because intestinal mucosal healing is closely related to the prognosis of IBD patients, and in the absence of means to promote mucosal healing, how to further improve the efficacy of current therapeutic measures and further develop new therapeutic modalities is a matter that needs to be considered at present and in the future. These new promising mucosal repair methods, including enteral nutrition, organoid transplantation, and intestinal microbiota transplantation, have been proposed, but the specific clinical practice remains a formidable challenge. Subsequent research will determine the viability of potentially promising pathways, while also weighing their impact against potential hazards and complexities like availability within biological systems, cell proliferation stimulation, and tumor formation.

## Author contributions

MW: Writing – original draft. JS: Writing – review & editing. CY: Writing – review & editing. XZ: Writing – review & editing. GX: Writing – review & editing. ZX: Writing – review & editing, Writing – original draft. YM: Writing – review & editing.

## References

[1] Agrawal M Spencer EA Colombel JF Ungaro RC. Approach to the management of recently diagnosed inflammatory bowel disease patients: A user's guide for adult and pediatric gastroenterologists. Gastroenterology (2021) 161(1):47–65. doi: 10.1053/j.gastro.2021.04.063 33940007 PMC8640961

[2] Kobayashi T Siegmund B Le Berre C Wei SC Ferrante M Shen B. Ulcerative colitis. Nat Rev Dis Primers (2020) 6(1):74. doi: 10.1038/s41572-020-0205-x 32913180

[3] Roda G Chien Ng S Kotze PG Argollo M Panaccione R Spinelli A. Crohn's disease. Nat Rev Dis Primers (2020) 6(1):22. doi: 10.1038/s41572-020-0156-2 32242028

[4] Pariente B Hu S Bettenworth D Speca S Desreumaux P Meuwis MA. Treatments for Crohn's disease-associated bowel damage: A systematic review. Clin Gastroenterol Hepatol (2019) 17(5):847–56. doi: 10.1016/j.cgh.2018.06.043 30012430

[5] Kaplan GG. The global burden of IBD: from 2015 to 2025. Nat Rev Gastroenterol Hepatol (2015) 12(12):720–7. doi: 10.1038/nrgastro.2015.150 26323879

[6] Gallo G Kotze PG Spinelli A. Surgery in ulcerative colitis: When? How? Best Pract Res Clin Gastroenterol (2018) 32-33:71–8. doi: 10.1016/j.bpg.2018.05.017 30060941

[7] Adamina M Bonovas S Raine T Spinelli A Warusavitarne J Armuzzi A. ECCO guidelines on therapeutics in Crohn's disease: surgical treatment. J Crohns Colitis (2020) 14(2):155–68. doi: 10.1093/ecco-jcc/jjz187 31742338

[8] Patel KV Darakhshan AA Griffin N Williams AB Sanderson JD Irving PM. Patient optimization for surgery relating to Crohn's disease. Nat Rev Gastroenterol Hepatol (2016) 13(12):707–19. doi: 10.1038/nrgastro.2016.158 27780971

[9] Ghouri YA Tahan V Shen B. Secondary causes of inflammatory bowel diseases. World J Gastroenterol (2020) 26:3998–4017. doi: 10.3748/wjg.v26.i28.3998 32821067 PMC7403802

[10] Nighot P Ma T. Endocytosis of intestinal tight junction proteins: in time and space. Inflammation Bowel Dis (2021) 27(2):283–90. doi: 10.1093/ibd/izaa141 PMC7813749 32497180

[11] Yuen J Pluthero FG Douda DN Riedl M Cherry A Ulanova M. NETosing neutrophils activate complement both on their own NETs and Bacteria via alternative and non-alternative pathways. Front Immunol (2016) 7:137. doi: 10.3389/fimmu.2016.00137 27148258 PMC4831636

[12] Mann EA Saeed SA. Gastrointestinal infection as a trigger for inflammatory bowel disease. Curr Opin Gastroenterol (2012) 28(1):24–9. doi: 10.1097/MOG.0b013e32834c453e 22080823

[13] Elinav E Strowig T Kau AL Henao-Mejia J Thaiss CA Booth CJ. NLRP6 inflammasome regulates colonic microbial ecology and risk for colitis. Cell (2011) 145(5):745–57. doi: 10.1016/j.cell.2011.04.022 PMC3140910 21565393

[14] Liang J Huang HI Benzatti FP Karlsson AB Zhang JJ Youssef N. Inflammatory Th1 and Th17 in the intestine are each driven by functionally specialized dendritic cells with distinct requirements for myD88. Cell Rep (2016) 17(5):1330–43. doi: 10.1016/j.celrep.2016.09.091 PMC5123685 27783947

[15] Frede A Czarnewski P Monasterio G Tripathi KP Bejarano DA Ramirez Flores RO. B cell expansion hinders the stroma-epithelium regenerative cross talk during mucosal healing. Immunity (2022) 55(12):2336–51.e12. doi: 10.1016/j.immuni.2022.11.002 36462502

[16] Otte ML Lama Tamang R Papapanagiotou J Ahmad R Dhawan P Singh AB. Mucosal healing and inflammatory bowel disease: Therapeutic implications and new targets. World J Gastroenterol (2023) 29(7):1157–72. doi: 10.3748/wjg.v29.i7.1157 PMC10011951 36926666

[17] Sinopoulou V Gordon M Akobeng AK Gasparetto M Sammaan M Vasiliou J. Interventions for the management of abdominal pain in Crohn's disease and inflammatory bowel disease. Cochrane Database Syst Rev (2021) 11(11):CD013531. doi: 10.1002/14651858.CD013531.pub2 34844288 PMC8629648

[18] Papay P Ignjatovic A Karmiris K Amarante H Milheller P Feagan B. Optimising monitoring in the management of Crohn's disease: a physician's perspective. J Crohns Colitis (2013) 7(8):653–69. doi: 10.1016/j.crohns.2013.02.005 23562672

[19] Gupta V Mohsen W Chapman TP Satsangi J. Predicting outcome in acute severe colitis-controversies in clinical practice in 2021. J Crohns Colitis (2021) 15(7):1211–21. doi: 10.1093/ecco-jcc/jjaa265 PMC7799290 33388777

[20] State M Negreanu L Voiosu T Voiosu A Balanescu P Mateescu RB. Surrogate markers of mucosal healing in inflammatory bowel disease: A systematic review. World J Gastroenterol (2021) 27(16):1828–40. doi: 10.3748/wjg.v27.i16.1828 PMC8072191 33967560

[21] Mazzuoli S Guglielmi FW Antonelli E Salemme M Bassotti G Villanacci V. Definition and evaluation of mucosal healing in clinical practice. Dig Liver Dis (2013) 45(12):969–77. doi: 10.1016/j.dld.2013.06.010 23932331

[22] Boal Carvalho P Cotter J. Mucosal healing in ulcerative colitis: A comprehensive review. Drugs (2017) 77(2):159–73. doi: 10.1007/s40265-016-0676-y 28078646

[23] Ramos GP Papadakis KA. Mechanisms of disease: inflammatory bowel diseases. Mayo Clin Proc (2019) 94(1):155–65. doi: 10.1016/j.mayocp.2018.09.013 PMC6386158 30611442

[24] Gomaa EZ. Human gut microbiota/microbiome in health and diseases: a review. Antonie Van Leeuwenhoek (2020) 113(12):2019–40. doi: 10.1007/s10482-020-01474-7 33136284

[25] Villablanca EJ Selin K Hedin CRH. Mechanisms of mucosal healing: treating inflammatory bowel disease without immunosuppression? Nat Rev Gastroenterol Hepatol (2022) 19(8):493–507. doi: 10.1038/s41575-022-00604-y 35440774

[26] Moriichi K Fujiya M Okumura T. The endoscopic diagnosis of mucosal healing and deep remission in inflammatory bowel disease. Dig Endosc (2021) 33(7):1008–23. doi: 10.1111/den.13863 33020947

[27] Daperno M D'Haens G Van Assche G Baert F Bulois P Maunoury V. Development and validation of a new, simplified endoscopic activity score for Crohn's disease: the SES-CD. Gastrointest Endosc (2004) 60(4):505–12. doi: 10.1016/s0016-5107(04)01878-4 15472670

[28] Barreiro-de Acosta M Vallejo N de la Iglesia D Uribarri L Bastón I Ferreiro-Iglesias R. Evaluation of the risk of relapse in ulcerative colitis according to the degree of mucosal healing (Mayo 0 vs 1): A longitudinal cohort study. J Crohns Colitis (2016) 10(1):13–9. doi: 10.1093/ecco-jcc/jjv158 26351390

[29] Pennelli G Grillo F Galuppini F Ingravallo G Pilozzi E Rugge M. Gastritis: update on etiological features and histological practical approach. Pathologica (2020) 112(3):153–65. doi: 10.32074/1591-951X-163 PMC7931571 33179619

[30] Magro F Sabino J Rosini F Tripathi M Borralho P Baldin P. ECCO position on harmonisation of Crohn's disease mucosal histopathology. J Crohns Colitis (2022) 16(6):876–83. doi: 10.1093/ecco-jcc/jjac006 35022677

[31] Marchal-Bressenot A Salleron J Boulagnon-Rombi C Bastien C Cahn V Cadiot G. Development and validation of the Nancy histological index for UC. Gut (2017) 66(1):43–9. doi: 10.1136/gutjnl-2015-310187 26464414

[32] Bryant RV Winer S Travis SP Riddell RH. Systematic review: histological remission in inflammatory bowel disease. Is 'complete' remission the new treatment paradigm? An IOIBD initiative. J Crohns Colitis (2014) 8(12):1582–97. doi: 10.1016/j.crohns.2014.08.011 25267173

[33] Caputo A Parente P Cadei M Fassan M Rispo A Leoncini G. Simplified Histologic Mucosal Healing Scheme (SHMHS) for inflammatory bowel disease: a nationwide multicenter study of performance and applicability. Tech Coloproctol (2022) 26(9):713–23. doi: 10.1007/s10151-022-02628-7 PMC9360061 35648263

[34] Gui X Bazarova A Del Amor R Vieth M de Hertogh G Villanacci V. PICaSSO Histologic Remission Index (PHRI) in ulcerative colitis: development of a novel simplified histological score for monitoring mucosal healing and predicting clinical outcomes and its applicability in an artificial intelligence system. Gut (2022) 71(5):889–98. doi: 10.1136/gutjnl-2021-326376 PMC8995819 35173041

[35] Denters MJ Schreuder M Depla AC Mallant-Hent RC van Kouwen MC Deutekom M. Patients' perception of colonoscopy: patients with inflammatory bowel disease and irritable bowel syndrome experience the largest burden. Eur J Gastroenterol Hepatol (2013) 25(8):964–72. doi: 10.1097/MEG.0b013e328361dcd3 23660935

[36] Kim SY Kim HS Park HJ. Adverse events related to colonoscopy: Global trends and future challenges. World J Gastroenterol (2019) 25(2):190–204. doi: 10.3748/wjg.v25.i2.190 30670909 PMC6337013

[37] Dragoni G Innocenti T Galli A. Biomarkers of inflammation in inflammatory bowel disease: how long before abandoning single-marker approaches? Dig Dis (2021) 39(3):190–203. doi: 10.1159/000511641 32942275

[38] Jukic A Bakiri L Wagner EF Tilg H Adolph TE. Calprotectin: from biomarker to biological function. Gut (2021) 70(10):1978–88. doi: 10.1136/gutjnl-2021-324855 PMC8458070 34145045

[39] Shinzaki S Matsuoka K Iijima H Mizuno S Serada S Fujimoto M. Leucine-rich Alpha-2 glycoprotein is a serum biomarker of mucosal healing in ulcerative colitis. J Crohns Colitis (2017) 11(1):84–91. doi: 10.1093/ecco-jcc/jjw132 27466171 PMC5175492

[40] Guo X Huang C Xu J Xu H Liu L Zhao H. Gut microbiota is a potential biomarker in inflammatory bowel disease. Front Nutr (2022) 8:818902. doi: 10.3389/fnut.2021.818902 35127797 PMC8814525

[41] Tarapatzi G Filidou E Kandilogiannakis L Vradelis S Kolios G. Biomarkers in inflammatory bowel diseases: predicting the indication and the effect of biologics. J Gastrointestin Liver Dis (2022) 31(2):229–43. doi: 10.15403/jgld-4229 35694983

[42] Koleth G Emmanue J Spadaccini M Mascagni P Khalaf K Mori Y. Artificial intelligence in gastroenterology: Where are we heading? Endosc Int Open (2022) 10(11):E1474–80. doi: 10.1055/a-1907-6569 PMC9666060 36397868

[43] Tontini GE Rimondi A Vernero M Neumann H Vecchi M Bezzio C. Artificial intelligence in gastrointestinal endoscopy for inflammatory bowel disease: a systematic review and new horizons. Therap Adv Gastroenterol (2021) 14:17562848211017730. doi: 10.1177/17562848211017730 PMC8202249 34178115

[44] Cooper ST McNeil PL. Membrane repair: mechanisms and pathophysiology. Physiol Rev (2015) 95(4):1205–40. doi: 10.1152/physrev.00037.2014 PMC4600952 26336031

[45] Okamoto R Watanabe M. Molecular and clinical basis for the regeneration of human gastrointestinal epithelia. J Gastroenterol (2004) 39(1):1–6. doi: 10.1007/s00535-003-1259-8 14767727

[46] Sturm A Dignass AU. Epithelial restitution and wound healing in inflammatory bowel disease. World J Gastroenterol (2008) 14(3):348–53. doi: 10.3748/wjg.14.348 PMC2679124 18200658

[47] Beck PL Rosenberg IM Xavier RJ Koh T Wong JF Podolsky DK. Transforming growth factor-beta mediates intestinal healing and susceptibility to injury *in vitro* and *in vivo* through epithelial cells. Am J Pathol (2003) 162(2):597–608. doi: 10.1016/s0002-9440(10)63853-9 12547717 PMC1851153

[48] Hoffmann P Zeeh JM Lakshmanan J Wu VS Procaccino F Reinshagen M. Increased expression of transforming growth factor alpha precursors in acute experimental colitis in rats. Gut (1997) 41(2):195–202. doi: 10.1136/gut.41.2.195 9301498 PMC1891469

[49] Oncel S Basson MD. Gut homeostasis, injury, and healing: New therapeutic targets. World J Gastroenterol (2022) 28(17):1725–50. doi: 10.3748/wjg.v28.i17.1725 PMC9099196 35633906

[50] Martini E Krug SM Siegmund B Neurath MF Becker C. Mend your fences: the epithelial barrier and its relationship with mucosal immunity in inflammatory bowel disease. Cell Mol Gastroenterol Hepatol (2017) 4(1):33–46. doi: 10.1016/j.jcmgh.2017.03.007 28560287 PMC5439240

[51] Singh S Feuerstein JD Binion DG Tremaine WJ. AGA technical review on the management of mild-to-moderate ulcerative colitis. Gastroenterology (2019) 156(3):769–808.e29. doi: 10.1053/j.gastro.2018.12.008 30576642 PMC6858923

[52] Magro F Cordeiro G Dias AM Estevinho MM. Inflammatory bowel disease - non-biological treatment. Pharmacol Res (2020) 160:105075. doi: 10.1016/j.phrs.2020.105075 32653651

[53] Ding Z Ninan K Johnston BC Moayyedi P Sherlock M Zachos M. Microbiota signatures and mucosal healing in the use of enteral nutrition therapy v. corticosteroids for the treatment of children with Crohn's disease: a systematic review and meta-analysis. Br J Nutr (2023) 15:1–18. doi: 10.1017/S0007114523000405 PMC10511686 36788671

[54] Granot M Cohen S Shouval DS Haberman Y Lahad A Assa A. Mucosal healing with methotrexate versus azathioprine treatment in pediatric Crohn's disease as reflected by fecal calprotectin. Minerva Pediatr (Torino) (2022). doi: 10.23736/S2724-5276.22.06745-3 35373937

[55] Picardo S Seow CH. The impact of pregnancy on biologic therapies for the treatment of inflammatory bowel disease. Best Pract Res Clin Gastroenterol (2020) 44-45:101670. doi: 10.1016/j.bpg.2020.101670 32359682

[56] Pugliese D Privitera G Crispino F Mezzina N Castiglione F Fiorino G. Effectiveness and safety of vedolizumab in a matched cohort of elderly and nonelderly patients with inflammatory bowel disease: the IG-IBD LIVE study. Aliment Pharmacol Ther (2022) 56(1):95–109. doi: 10.1111/apt.16923 35876062 PMC9324100

[57] Sommer K Wiendl M Müller TM Heidbreder K Voskens C Neurath MF. Intestinal mucosal wound healing and barrier integrity in IBD-crosstalk and trafficking of cellular players. Front Med (Lausanne) (2021) 8:643973. doi: 10.3389/fmed.2021.643973 33834033 PMC8021701

[58] Ungar B Levy I Yavne Y Yavzori M Picard O Fudim E. Optimizing anti-TNF-α Therapy: serum levels of infliximab and adalimumab are associated with mucosal healing in patients with inflammatory bowel diseases. Clin Gastroenterol Hepatol (2016) 14(4):550–7.e2. doi: 10.1016/j.cgh.2015.10.025 26538204

[59] Martin JC Chang C Boschetti G Ungaro R Giri M Grout JA. Single-cell analysis of Crohn's disease lesions identifies a pathogenic cellular module associated with resistance to anti-TNF therapy. Cell (2019) 178(6):1493–508.e20. doi: 10.1016/j.cell.2019.08.008 31474370 PMC7060942

[60] Rutgeerts P Gasink C Chan D Lang Y Pollack P Colombel JF. Efficacy of ustekinumab for inducing endoscopic healing in patients with Crohn's disease. Gastroenterology (2018) 155(4):1045–58. doi: 10.1053/j.gastro.2018.06.035 29909019

[61] D'Haens G Panaccione R Baert F Bossuyt P Colombel JF Danese S. Risankizumab as induction therapy for Crohn's disease: results from the phase 3 ADVANCE and MOTIVATE induction trials. Lancet (2022) 399(10340):2015–30. doi: 10.1016/S0140-6736(22)00467-6 35644154

[62] Peyrin-Biroulet L Allegretti JR Rubin DT Bressler B Germinaro M Huang KG. Guselkumab in patients with moderately to severely active ulcerative colitis: QUASAR phase 2b induction study. Gastroenterology (2023) 165(6):1443–57. doi: 10.1053/j.gastro.2023.08.038 37659673

[63] D'Haens G Dubinsky M Kobayashi T Irving PM Howaldt S Pokrotnieks J. Mirikizumab as induction and maintenance therapy for ulcerative colitis. N Engl J Med (2023) 388(26):2444–55. doi: 10.1056/NEJMoa2207940 37379135

[64] Feagan BG Rutgeerts P Sands BE Hanauer S Colombel JF Sandborn WJ. Vedolizumab as induction and maintenance therapy for ulcerative colitis. N Engl J Med (2013) 369(8):699–710. doi: 10.1056/NEJMoa1215734 23964932

[65] Nielsen OH Boye TL Gubatan J Chakravarti D Jaquith JB LaCasse EC. Selective JAK1 inhibitors for the treatment of inflammatory bowel disease. Pharmacol Ther (2023) 245:108402. doi: 10.1016/j.pharmthera.2023.108402 37004800

[66] Danese S Panaccione R Abreu MT Rubin DT Ghosh S Dignass A. Efficacy and safety of approximately 3 years of continuous ozanimod in moderately to severely active ulcerative colitis: interim analysis of the True North open-label extension. J Crohns Colitis (2023), jjad146. doi: 10.1093/ecco-jcc/jjad146 37651686 PMC10896634

[67] Raine T Ishiguro Y Rubin DT Finney-Hayward T Vladea R Liu J. Impact of baseline corticosteroid use on the efficacy and safety of upadacitinib in patients with ulcerative colitis: a post Hoc analysis of the phase 3 clinical trial programme. J Crohns Colitis (2023), jjad190. doi: 10.1093/ecco-jcc/jjad190 37942921 PMC11140624

[68] Pugliese D Felice C Papa A Gasbarrini A Rapaccini GL Guidi L. Anti TNF-α therapy for ulcerative colitis: current status and prospects for the future. Expert Rev Clin Immunol (2017) 13(3):223–33. doi: 10.1080/1744666X.2017.1243468 27687496

[69] Takeuchi I Kaburaki Y Arai K Shimizu H Hirano Y Nagata S. Infliximab for very early-onset inflammatory bowel disease: A tertiary center experience in Japan. J Gastroenterol Hepatol (2020) 35(4):593–600. doi: 10.1111/jgh.14836 31425641

[70] Sands BE Irving PM Hoops T Izanec JL Gao LL Gasink C. Ustekinumab versus adalimumab for induction and maintenance therapy in biologic-naive patients with moderately to severely active Crohn's disease: a multicentre, randomised, double-blind, parallel-group, phase 3b trial. Lancet (2022) 399(10342):2200–11. doi: 10.1016/S0140-6736(22)00688-2 35691323

[71] Cholapranee A Hazlewood GS Kaplan GG Peyrin-Biroulet L Ananthakrishnan AN. Systematic review with meta-analysis: comparative efficacy of biologics for induction and maintenance of mucosal healing in Crohn's disease and ulcerative colitis controlled trials. Aliment Pharmacol Ther (2017) 45(10):1291–302. doi: 10.1111/apt.14030 PMC5395316 28326566

[72] Dahmus J Rosario M Clarke K. Risk of lymphoma associated with anti-TNF therapy in patients with inflammatory bowel disease: implications for therapy. Clin Exp Gastroenterol (2020) 13:339–50. doi: 10.2147/CEG.S237646 PMC7501969 32982364

[73] Fine S Papamichael K Cheifetz AS. Etiology and management of lack or loss of response to anti-tumor necrosis factor therapy in patients with inflammatory bowel disease. Gastroenterol Hepatol (N Y) (2019) 15(12):656–65. PMC6935028 31892912

[74] Carroll MW Kuenzig ME Mack DR Otley AR Griffiths AM Kaplan GG. The impact of inflammatory bowel disease in Canada 2018: children and adolescents with IBD. J Can Assoc Gastroenterol (2019) 2(Suppl 1):S49–67. doi: 10.1093/jcag/gwy056 PMC6512244 31294385

[75] Forbes A Escher J Hébuterne X Kłęk S Krznaric Z Schneider S. ESPEN guideline: Clinical nutrition in inflammatory bowel disease. Clin Nutr (2017) 36(2):321–47. doi: 10.1016/j.clnu.2016.12.027 28131521

[76] Weingarden AR Vaughn BP. Intestinal microbiota, fecal microbiota transplantation, and inflammatory bowel disease. Gut Microbes (2017) 8(3):238–52. doi: 10.1080/19490976.2017.1290757 PMC5479396 28609251

[77] Krishnan K Arnone B Buchman A. Intestinal growth factors: potential use in the treatment of inflammatory bowel disease and their role in mucosal healing. Inflammation Bowel Dis (2011) 17(1):410–22. doi: 10.1002/ibd.21316 20848489

[78] Ananthakrishnan AN Khalili H Song M Higuchi LM Richter JM Nimptsch K. High school diet and risk of Crohn's disease and ulcerative colitis. Inflammation Bowel Dis (2015) 21(10):2311–9. doi: 10.1097/MIB.0000000000000501 PMC4567521 26236952

[79] Reddavide R Rotolo O Caruso MG Stasi E Notarnicola M Miraglia C. The role of diet in the prevention and treatment of Inflammatory Bowel Diseases. Acta Biomed (2018) 89(9-S):60–75. doi: 10.23750/abm.v89i9-S.7952 PMC6502201 30561397

[80] Urlep D Orel R Kunstek P Benedik E. Treatment of active Crohn's disease in children using partial enteral nutrition combined with a modified Crohn's disease exclusion diet: A pilot prospective cohort trial on clinical and endoscopic outcomes. Nutrients (2023) 15(21):4676. doi: 10.3390/nu15214676 37960328 PMC10650058

[81] Olendzki BC Silverstein TD Persuitte GM Ma Y Baldwin KR Cave D. An anti-inflammatory diet as treatment for inflammatory bowel disease: a case series report. Nutr J (2014) 13:5. doi: 10.1186/1475-2891-13-5 24428901 PMC3896778

[82] Rivera N Nguyen K Kalami V Qin F Mathur MB Blankenburg R. A specific carbohydrate diet virtual teaching kitchen curriculum promotes knowledge and confidence in caregivers of pediatric patients with inflammatory bowel disease. Nutrients (2023) 15(18):3999. doi: 10.3390/nu15183999 37764781 PMC10537188

[83] Sasson AN Ananthakrishnan AN Raman M. Diet in treatment of inflammatory bowel diseases. Clin Gastroenterol Hepatol (2021) 19(3):425–35.e3. doi: 10.1016/j.cgh.2019.11.054 31812656

[84] Ruemmele FM Veres G Kolho KL Griffiths A Levine A Escher JC. Consensus guidelines of ECCO/ESPGHAN on the medical management of pediatric Crohn's disease. J Crohns Colitis (2014) 8(10):1179–207. doi: 10.1016/j.crohns.2014.04.005 24909831

[85] Borrelli O Cordischi L Cirulli M Paganelli M Labalestra V Uccini S. Polymeric diet alone versus corticosteroids in the treatment of active pediatric Crohn's disease: a randomized controlled open-label trial. Clin Gastroenterol Hepatol (2006) 4(6):744–53. doi: 10.1016/j.cgh.2006.03.010 16682258

[86] Pigneur B Lepage P Mondot S Schmitz J Goulet O Doré J. Mucosal healing and bacterial composition in response to enteral nutrition vs steroid-based induction therapy-A randomised prospective clinical trial in children with Crohn's disease. J Crohns Colitis (2019) 13(7):846–55. doi: 10.1093/ecco-jcc/jjy207 30541015

[87] Wall CL Day AS Gearry RB. Use of exclusive enteral nutrition in adults with Crohn's disease: a review. World J Gastroenterol (2013) 19(43):7652–60. doi: 10.3748/wjg.v19.i43.7652 PMC3837264 24282355

[88] Wall CL Gearry RB Day AS. Treatment of active Crohn's disease with exclusive and partial enteral nutrition: A pilot study in adults. Inflammation Intest Dis (2018) 2(4):219–27. doi: 10.1159/000489630 PMC6135224 30221149

[89] Yang Q Gao X Chen H Li M Wu X Zhi M. Efficacy of exclusive enteral nutrition in complicated Crohn's disease. Scand J Gastroenterol (2017) 52(9):995–1001. doi: 10.1080/00365521.2017.1335770 28598298

[90] Sahu P Kedia S Vuyyuru SK Bajaj A Markandey M Singh N. Randomised clinical trial: exclusive enteral nutrition versus standard of care for acute severe ulcerative colitis. Aliment Pharmacol Ther (2021) 53(5):568–76. doi: 10.1111/apt.16249 33440046

[91] Gerasimidis K Bertz M Hanske L Junick J Biskou O Aguilera M. Decline in presumptively protective gut bacterial species and metabolites are paradoxically associated with disease improvement in pediatric Crohn's disease during enteral nutrition. Inflammation Bowel Dis (2014) 20(5):861–71. doi: 10.1097/MIB.0000000000000023 24651582

[92] Diederen K Li JV Donachie GE de Meij TG de Waart DR Hakvoort TBM. Exclusive enteral nutrition mediates gut microbial and metabolic changes that are associated with remission in children with Crohn's disease. Sci Rep (2020) 10(1):18879. doi: 10.1038/s41598-020-75306-z 33144591 PMC7609694

[93] Bannerjee K Camacho-Hübner C Babinska K Dryhurst KM Edwards R Savage MO. Anti-inflammatory and growth-stimulating effects precede nutritional restitution during enteral feeding in Crohn disease. J Pediatr Gastroenterol Nutr (2004) 38(3):270–5. doi: 10.1097/00005176-200403000-00007 15076624

[94] Tuveson D Clevers H. Cancer modeling meets human organoid technology. Science (2019) 364(6444):952–5. doi: 10.1126/science.aaw6985 31171691

[95] Sprangers J Zaalberg IC Maurice MM. Organoid-based modeling of intestinal development, regeneration, and repair. Cell Death Differ (2021) 28(1):95–107. doi: 10.1038/s41418-020-00665-z 33208888 PMC7852609

[96] Sato T Vries RG Snippert HJ van de Wetering M Barker N Stange DE. Single Lgr5 stem cells build crypt-villus structures *in vitro* without a mesenchymal niche. Nature (2009) 459(7244):262–5. doi: 10.1038/nature07935 19329995

[97] Lucafò M Muzzo A Marcuzzi M Giorio L Decorti G Stocco G. Patient-derived organoids for therapy personalization in inflammatory bowel diseases. World J Gastroenterol (2022) 28(24):2636–53. doi: 10.3748/wjg.v28.i24.2636 PMC9260862 35979165

[98] Watanabe S Nishimura R Shirasaki T Katsukura N Hibiya S Kirimura S. Schlafen 11 is a novel target for mucosal regeneration in ulcerative colitis. J Crohns Colitis (2021) 15(9):1558–72. doi: 10.1093/ecco-jcc/jjab032 33596306

[99] Jee J Jeong SY Kim HK Choi SY Jeong S Lee J. *In vivo* evaluation of scaffolds compatible for colonoid engraftments onto injured mouse colon epithelium. FASEB J (2019) 33(9):10116–25. doi: 10.1096/fj.201802692RR 31211931

[100] Yui S Nakamura T Sato T Nemoto Y Mizutani T Zheng X. Functional engraftment of colon epithelium expanded *in vitro* from a single adult Lgr5^+^ stem cell. Nat Med (2012) 18(4):618–23. doi: 10.1038/nm.2695 22406745

[101] Sugimoto S Ohta Y Fujii M Matano M Shimokawa M Nanki K. Reconstruction of the human colon epithelium *in vivo* . Cell Stem Cell (2018) 22(2):171–6.e5. doi: 10.1016/j.stem.2017.11.012 29290616

[102] Watanabe S Kobayashi S Ogasawara N Okamoto R Nakamura T Watanabe M. Transplantation of intestinal organoids into a mouse model of colitis. Nat Protoc (2022) 17(3):649–71. doi: 10.1038/s41596-021-00658-3 35110738

[103] Yoo JH Donowitz M. Intestinal enteroids/organoids: A novel platform for drug discovery in inflammatory bowel diseases. World J Gastroenterol (2019) 25(30):4125–47. doi: 10.3748/wjg.v25.i30.4125 PMC6700704 31435168

[104] Grégoire C Lechanteur C Briquet A Baudoux É Baron F Louis E. Review article: mesenchymal stromal cell therapy for inflammatory bowel diseases. Aliment Pharmacol Ther (2017) 45(2):205–21. doi: 10.1111/apt.13864 27878827

[105] Barker N. Adult intestinal stem cells: critical drivers of epithelial homeostasis and regeneration. Nat Rev Mol Cell Biol (2014) 15(1):19–33. doi: 10.1038/nrm3721 24326621

[106] Saadh MJ Mikhailova MV Rasoolzadegan S Falaki M Akhavanfar R Gonzáles JLA. Therapeutic potential of mesenchymal stem/stromal cells (MSCs)-based cell therapy for inflammatory bowel diseases (IBD) therapy. Eur J Med Res (2023) 28(1):47. doi: 10.1186/s40001-023-01008-7 36707899 PMC9881387

[107] Wang L Deng Z Sun Y Zhao Y Li Y Yang M. The study on the regulation of Th cells by mesenchymal stem cells through the JAK-STAT signaling pathway to protect naturally aged sepsis model rats. Front Immunol (2022) 13:820685. doi: 10.3389/fimmu.2022.820685 35197984 PMC8858840

[108] Hashemi SM Hassan ZM Hossein-Khannazer N Pourfathollah AA Soudi S. Investigating the route of administration and efficacy of adipose tissue-derived mesenchymal stem cells and conditioned medium in type 1 diabetic mice. Inflammopharmacology (2020) 28(2):585–601. doi: 10.1007/s10787-019-00661-x 31741175

[109] Lightner AL Dozois EJ Dietz AB Fletcher JG Friton J Butler G. Matrix-delivered autologous mesenchymal stem cell therapy for refractory rectovaginal Crohn's fistulas. Inflammation Bowel Dis (2020) 26(5):670–7. doi: 10.1093/ibd/izz215 31605115

[110] Panés J García-Olmo D Van Assche G Colombel JF Reinisch W Baumgart DC. Expanded allogeneic adipose-derived mesenchymal stem cells (Cx601) for complex perianal fistulas in Crohn's disease: a phase 3 randomised, double-blind controlled trial. Lancet (2016) 388(10051):1281–90. doi: 10.1016/S0140-6736(16)31203-X 27477896

[111] Shi X Chen Q Wang F. Mesenchymal stem cells for the treatment of ulcerative colitis: a systematic review and meta-analysis of experimental and clinical studies. Stem Cell Res Ther (2019) 10(1):266. doi: 10.1186/s13287-019-1336-4 31443677 PMC6708175

[112] Forbes GM Sturm MJ Leong RW Sparrow MP Segarajasingam D Cummins AG. A phase 2 study of allogeneic mesenchymal stromal cells for luminal Crohn's disease refractory to biologic therapy. Clin Gastroenterol Hepatol (2014) 12(1):64–71. doi: 10.1016/j.cgh.2013.06.021 23872668

[113] Dhere T Copland I Garcia M Chiang KY Chinnadurai R Prasad M. The safety of autologous and metabolically fit bone marrow mesenchymal stromal cells in medically refractory Crohn's disease - a phase 1 trial with three doses. Aliment Pharmacol Ther (2016) 44(5):471–81. doi: 10.1111/apt.13717 27385373

[114] Sala E Genua M Petti L Anselmo A Arena V Cibella J. Mesenchymal stem cells reduce colitis in mice via release of TSG6, independently of their localization to the intestine. Gastroenterology (2015) 149(1):163–76. doi: 10.1053/j.gastro.2015.03.013 25790743

[115] Wang Y Huang B Jin T Ocansey DKW Jiang J Mao F. Intestinal fibrosis in inflammatory bowel disease and the prospects of mesenchymal stem cell therapy. Front Immunol (2022) 13:835005. doi: 10.3389/fimmu.2022.835005 35370998 PMC8971815

[116] Zhang Y Chen J Fu H Kuang S He F Zhang M. Exosomes derived from 3D-cultured MSCs improve therapeutic effects in periodontitis and experimental colitis and restore the Th17 cell/Treg balance in inflamed periodontium. Int J Oral Sci (2021) 13(1):43. doi: 10.1038/s41368-021-00150-4 34907166 PMC8671433

[117] Yang S Liang X Song J Li C Liu A Luo Y. A novel therapeutic approach for inflammatory bowel disease by exosomes derived from human umbilical cord mesenchymal stem cells to repair intestinal barrier via TSG-6. Stem Cell Res Ther (2021) 12(1):315. doi: 10.1186/s13287-021-02404-8 34051868 PMC8164818

[118] Song EM Joo YH Choe AR Park Y Tae CH Hong JT. Three-dimensional culture method enhances the therapeutic efficacies of tonsil-derived mesenchymal stem cells in murine chronic colitis model. Sci Rep (2021) 11(1):19589. doi: 10.1038/s41598-021-98711-4 34599237 PMC8486762

[119] Nishida A Nishino K Sakai K Owaki Y Noda Y Imaeda H. Can control of gut microbiota be a future therapeutic option for inflammatory bowel disease? World J Gastroenterol (2021) 27(23):3317–26. doi: 10.3748/wjg.v27.i23.3317 PMC8218353 34163114

[120] Frank DN St Amand AL Feldman RA Boedeker EC Harpaz N Pace NR. Molecular-phylogenetic characterization of microbial community imbalances in human inflammatory bowel diseases. Proc Natl Acad Sci U.S.A. (2007) 104(34):13780–5. doi: 10.1073/pnas.0706625104 PMC1959459 17699621

[121] Morgan XC Tickle TL Sokol H Gevers D Devaney KL Ward DV. Dysfunction of the intestinal microbiome in inflammatory bowel disease and treatment. Genome Biol (2012) 13(9):R79. doi: 10.1186/gb-2012-13-9-r79 23013615 PMC3506950

[122] Parada Venegas D de la Fuente MK Landskron G González MJ Quera R Dijkstra G. Short chain fatty acids (SCFAs)-mediated gut epithelial and immune regulation and its relevance for inflammatory bowel diseases. Front Immunol (2019) 10:277. doi: 10.3389/fimmu.2019.00277 30915065 PMC6421268

[123] Nishida A Inoue R Inatomi O Bamba S Naito Y Andoh A. Gut microbiota in the pathogenesis of inflammatory bowel disease. Clin J Gastroenterol (2018) 11(1):1–10. doi: 10.1007/s12328-017-0813-5 29285689

[124] Chen ML Ge Z Fox JG Schauer DB. Disruption of tight junctions and induction of proinflammatory cytokine responses in colonic epithelial cells by Campylobacter jejuni. Infect Immun (2006) 74(12):6581–9. doi: 10.1128/IAI.00958-06 PMC1698078 17015453

[125] Imdad A Pandit NG Zaman M Minkoff NZ Tanner-Smith EE Gomez-Duarte OG. Fecal transplantation for treatment of inflammatory bowel disease. Cochrane Database Syst Rev (2023) 4(4):CD012774. doi: 10.1002/14651858.CD012774.pub3 37094824 PMC10133790

[126] Alam A Leoni G Quiros M Wu H Desai C Nishio H. The microenvironment of injured murine gut elicits a local pro-restitutive microbiota. Nat Microbiol (2016) 1:15021. doi: 10.1038/nmicrobiol.2015.21 27571978 PMC5076466

[127] Bessman NJ Mathieu JRR Renassia C Zhou L Fung TC Fernandez KC. Dendritic cell-derived hepcidin sequesters iron from the microbiota to promote mucosal healing. Science (2020) 368(6487):186–9. doi: 10.1126/science.aau6481 PMC7724573 32273468

[128] Liang L Liu L Zhou W Yang C Mai G Li H. Gut microbiota-derived butyrate regulates gut mucus barrier repair by activating the macrophage/WNT/ERK signaling pathway. Clin Sci (Lond) (2022) 136(4):291–307. doi: 10.1042/CS20210778 35194640

[129] Zou B Liu S Li X He J Dong C Ruan M. Repeated and multiple fecal microbiota transplantations plus partial enteral nutrition as the first-line treatment in active pediatric Crohn's disease. Front Cell Infect Microbiol (2023) 13:1083236. doi: 10.3389/fcimb.2023.1083236 36909725 PMC9996013

[130] Kunde S Pham A Bonczyk S Crumb T Duba M Conrad H Jr. Safety, tolerability, and clinical response after fecal transplantation in children and young adults with ulcerative colitis. J Pediatr Gastroenterol Nutr (2013) 56(6):597–601. doi: 10.1097/MPG.0b013e318292fa0d 23542823

[131] Quera R Espinoza R Estay C Rivera D. Bacteremia as an adverse event of fecal microbiota transplantation in a patient with Crohn's disease and recurrent Clostridium difficile infection. J Crohns Colitis (2014) 8(3):252–3. doi: 10.1016/j.crohns.2013.10.002 24184170

[132] Marcella C Cui B Kelly CR Ianiro G Cammarota G Zhang F. Systematic review: the global incidence of faecal microbiota transplantation-related adverse events from 2000 to 2020. Aliment Pharmacol Ther (2021) 53(1):33–42. doi: 10.1111/apt.16148 33159374

[133] Lee C Louie T Bancke L Guthmueller B Harvey A Feuerstadt P. Safety of fecal microbiota, live-jslm (REBYOTA) in individuals with recurrent Clostridioides difficile infection: data from five prospective clinical trials. Therap Adv Gastroenterol (2023) 16:17562848231174277. doi: 10.1177/17562848231174277 PMC10272687 37333464

[134] Jain N Umar TP Fahner AF Gibietis V. Advancing therapeutics for recurrent clostridioides difficile infections: an overview of vowst's FDA approval and implications. Gut Microbes (2023) 15(1):2232137. doi: 10.1080/19490976.2023.2232137 37431860 PMC10337487

[135] Zhang Z Zhang H Chen T Shi L Wang D Tang D. Regulatory role of short-chain fatty acids in inflammatory bowel disease. Cell Commun Signal (2022) 20(1):64. doi: 10.1186/s12964-022-00869-5 35546404 PMC9097439

[136] Corrêa-Oliveira R Fachi JL Vieira A Sato FT Vinolo MA. Regulation of immune cell function by short-chain fatty acids. Clin Transl Immunol (2016) 5(4):e73. doi: 10.1038/cti.2016.17 PMC4855267 27195116

[137] De Preter V Arijs I Windey K Vanhove W Vermeire S Schuit F. Impaired butyrate oxidation in ulcerative colitis is due to decreased butyrate uptake and a defect in the oxidation pathway. Inflammation Bowel Dis (2012) 18(6):1127–36. doi: 10.1002/ibd.21894 21987487

[138] Zhang D Jian YP Zhang YN Li Y Gu LT Sun HH. Short-chain fatty acids in diseases. Cell Commun Signal (2023) 21(1):212. doi: 10.1186/s12964-023-01219-9 37596634 PMC10436623

[139] Laffin M Fedorak R Zalasky A Park H Gill A Agrawal A. A high-sugar diet rapidly enhances susceptibility to colitis via depletion of luminal short-chain fatty acids in mice. Sci Rep (2019) 9(1):12294. doi: 10.1038/s41598-019-48749-2 31444382 PMC6707253

[140] Facchin S Vitulo N Calgaro M Buda A Romualdi C Pohl D. Microbiota changes induced by microencapsulated sodium butyrate in patients with inflammatory bowel disease. Neurogastroenterol Motil (2020) 32(10):e13914. doi: 10.1111/nmo.13914 32476236 PMC7583468

[141] Gelse K Pöschl E Aigner T. Collagens–structure, function, and biosynthesis. Adv Drug Delivery Rev (2003) 55(12):1531–46. doi: 10.1016/j.addr.2003.08.002 14623400

[142] Shimshoni E Yablecovitch D Baram L Dotan I Sagi I. ECM remodelling in IBD: innocent bystander or partner in crime? The emerging role of extracellular molecular events in sustaining intestinal inflammation. Gut (2015) 64(3):367–72. doi: 10.1136/gutjnl-2014-308048 PMC4345769 25416065

[143] Mortensen JH Lindholm M Langholm LL Kjeldsen J Bay-Jensen AC Karsdal MA. The intestinal tissue homeostasis - the role of extracellular matrix remodeling in inflammatory bowel disease. Expert Rev Gastroenterol Hepatol (2019) 13(10):977–93. doi: 10.1080/17474124.2019.1673729 31587588

[144] Frantz C Stewart KM Weaver VM. The extracellular matrix at a glance. J Cell Sci (2010) 123(Pt 24):4195–200. doi: 10.1242/jcs.023820 PMC2995612 21123617

[145] Stramer BM Mori R Martin P. The inflammation-fibrosis link? A Jekyll and Hyde role for blood cells during wound repair. J Invest Dermatol (2007) 127(5):1009–17. doi: 10.1038/sj.jid.5700811 17435786

[146] Petrey AC de la Motte CA. The extracellular matrix in IBD: a dynamic mediator of inflammation. Curr Opin Gastroenterol (2017) 33(4):234–8. doi: 10.1097/MOG.0000000000000368 PMC5562400 28562487

[147] Lindholm M Manon-Jensen T Madsen GI Krag A Karsdal MA Kjeldsen J. Extracellular matrix fragments of the basement membrane and the interstitial matrix are serological markers of intestinal tissue remodeling and disease activity in dextran sulfate sodium colitis. Dig Dis Sci (2019) 64(11):3134–42. doi: 10.1007/s10620-019-05676-6 31123972

[148] Göke M Zuk A Podolsky DK. Regulation and function of extracellular matrix intestinal epithelial restitution *in vitro* . Am J Physiol (1996) 271(5 Pt 1):G729–40. doi: 10.1152/ajpgi.1996.271.5.G729 8944685

[149] Stronati L Palone F Negroni A Colantoni E Mancuso AB Cucchiara S. Dipotassium glycyrrhizate improves intestinal mucosal healing by modulating extracellular matrix remodeling genes and restoring epithelial barrier functions. Front Immunol (2019) 10:939. doi: 10.3389/fimmu.2019.00939 31105713 PMC6498413

[150] Derkacz A Olczyk P Olczyk K Komosinska-Vassev K. The role of extracellular matrix components in inflammatory bowel diseases. J Clin Med (2021) 10(5):1122. doi: 10.3390/jcm10051122 33800267 PMC7962650

[151] Ferdowsi Khosroshahi A Soleimani Rad J Kheirjou R Roshangar B Rashtbar M Salehi R. Adipose tissue-derived stem cells upon decellularized ovine small intestine submucosa for tissue regeneration: An optimization and comparison method. J Cell Physiol (2020) 235(2):1556–67. doi: 10.1002/jcp.29074 31400002

[152] Chameettachal S Venuganti A Parekh Y Prasad D Joshi VP Vashishtha A. Human cornea-derived extracellular matrix hydrogel for prevention of post-traumatic corneal scarring: A translational approach. Acta Biomater (2023) 171:289–307. doi: 10.1016/j.actbio.2023.09.002 37683964

[153] Nakamura T Sato T. Advancing intestinal organoid technology toward regenerative medicine. Cell Mol Gastroenterol Hepatol (2017) 5(1):51–60. doi: 10.1016/j.jcmgh.2017.10.006 29204508 PMC5704126

[154] Wu MY Hill CS. Tgf-beta superfamily signaling in embryonic development and homeostasis. Dev Cell (2009) 16:329–43. doi: 10.1016/j.devcel.2009.02.012 19289080

[155] Di Sabatino A Pickard KM Rampton D Kruidenier L Rovedatti L Leakey NA. Blockade of transforming growth factor beta upregulates T-box transcription factor T-bet, and increases T helper cell type 1 cytokine and matrix metalloproteinase-3 production in the human gut mucosa. Gut (2008) 57(5):605–12. doi: 10.1136/gut.2007.130922 18178611

[156] Monteleone G Kumberova A Croft NM McKenzie C Steer HW MacDonald TT. Blocking Smad7 restores TGF-beta1 signaling in chronic inflammatory bowel disease. J Clin Invest (2001) 108(4):601–9. doi: 10.1172/JCI12821 PMC209401 11518734

[157] Ben-Lulu S Pollak Y Mogilner J Bejar J G Coran A Sukhotnik I. Dietary transforming growth factor-beta 2 (TGF-β2) supplementation reduces methotrexate-induced intestinal mucosal injury in a rat. PloS One (2012) 7(9):e45221. doi: 10.1371/journal.pone.0045221 22984629 PMC3440324

[158] di Mola FF Friess H Scheuren A Di Sebastiano P Graber H Egger B. Transforming growth factor-betas and their signaling receptors are coexpressed in Crohn's disease. Ann Surg (1999) 229(1):67–75. doi: 10.1097/00000658-199901000-00009 9923802 PMC1191610

[159] Monteleone G Neurath MF Ardizzone S Di Sabatino A Fantini MC Castiglione F. Mongersen, an oral SMAD7 antisense oligonucleotide, and Crohn's disease. N Engl J Med (2015) 372(12):1104–13. doi: 10.1056/NEJMoa1407250 25785968

[160] Monteleone G Di Sabatino A Ardizzone S Pallone F Usiskin K Zhan X. Impact of patient characteristics on the clinical efficacy of mongersen (GED-0301), an oral Smad7 antisense oligonucleotide, in active Crohn's disease. Aliment Pharmacol Ther (2016) 43(6):717–24. doi: 10.1111/apt.13526 PMC4849204 26766141

[161] Sands BE Feagan BG Sandborn WJ Schreiber S Peyrin-Biroulet L Frédéric Colombel J. Mongersen (GED-0301) for active Crohn's disease: results of a phase 3 study. Am J Gastroenterol (2020) 115(5):738–45. doi: 10.14309/ajg.0000000000000493 31850931

[162] Monteleone G Stolfi C Marafini I Atreya R Neurath MF. Smad7 antisense oligonucleotide-based therapy in Crohn's disease: is it time to re-evaluate? Mol Diagn Ther (2022) 26(5):477–81. doi: 10.1007/s40291-022-00606-1 PMC9411088 35841457

[163] Schuler C Foti F Perren L Mamie C Weder B Stokmaier M. Deletion of Smad7 ameliorates intestinal inflammation and contributes to fibrosis. Inflammation Bowel Dis (2023) 29(4):647–60. doi: 10.1093/ibd/izac221 36282601

[164] Aamann L Vestergaard EM Grønbæk H. Trefoil factors in inflammatory bowel disease. World J Gastroenterol (2014) 20(12):3223–30. doi: 10.3748/wjg.v20.i12.3223 PMC3964394 24696606

[165] Aihara E Engevik KA Montrose MH. Trefoil factor peptides and gastrointestinal function. Annu Rev Physiol (2017) 79:357–80. doi: 10.1146/annurev-physiol-021115-105447 PMC5474939 27992733

[166] Sun Z Liu H Yang Z Shao D Zhang W Ren Y. Intestinal trefoil factor activates the PI3K/Akt signaling pathway to protect gastric mucosal epithelium from damage. Int J Oncol (2014) 45(3):1123–32. doi: 10.3892/ijo.2014.2527 24990304

[167] Teng X Yang Y Liu L Yang L Wu J Sun M. Evaluation of inflammatory bowel disease activity in children using serum trefoil factor peptide. Pediatr Res (2020) 88(5):792–5. doi: 10.1038/s41390-020-0812-y 32120375

[168] Kjellev S Thim L Pyke C Poulsen SS. Cellular localization, binding sites, and pharmacologic effects of TFF3 in experimental colitis in mice. Dig Dis Sci (2007) 52(4):1050–9. doi: 10.1007/s10620-006-9256-4 17342398

[169] Poulsen SS Kissow H Hare K Hartmann B Thim L. Luminal and parenteral TFF2 and TFF3 dimer and monomer in two models of experimental colitis in the rat. Regul Pept (2005) 126(3):163–71. doi: 10.1016/j.regpep.2004.09.007 15664663

[170] Babyatsky MW deBeaumont M Thim L Podolsky DK. Oral trefoil peptides protect against ethanol- and indomethacin-induced gastric injury in rats. Gastroenterology (1996) 110(2):489–97. doi: 10.1053/gast.1996.v110.pm8566596 8566596

[171] Sun Y Zhu Y Wang L Mao X Peng X Peng Y. Recombinant adenovirus-mediated intestinal trefoil factor gene therapy for burn-induced intestinal mucosal injury. PLoS One (2013) 8(4):e62429. doi: 10.1371/journal.pone.0062429 23638081 PMC3634752

